# When Pedagogy Is Painful: Teaching in Tumultuous Times

**DOI:** 10.1177/0092055X17754120

**Published:** 2018-04

**Authors:** Marisela Martinez-Cola, with, Rocco English, Jennifer Min, Jonathan Peraza, Jamesetta Tambah, Christina Yebuah

**Affiliations:** 1 Emory University, Atlanta, GA, USA

**Keywords:** race and ethnicity, emotions and teaching, student engagement

## Abstract

What happens when the outside world begins to affect the classroom? Is the classroom supposed to be neutral, objective, and devoid of feelings? Or is it a space where students and teacher meet for healing, understanding, and critical thinking? From news reports of police brutality to highly publicized acts of racial aggression, students are inundated with examples of intolerance, hatred, and racial inequality. Those committed to critical pedagogy and social justice invite, embrace, and use these events to enhance classroom materials. What happens, however, when pedagogy is painful for both the student and the teacher? Several articles address the teacher’s experience and others the student experience. This article is dedicated to synthesizing and discussing both experiences from one course, Race and Ethnicity, at a height of racial tensions in the United States and on campus and providing the personal and pedagogical strategies that developed from the course.

As a Latina who identifies as a Muxerista,^1^ I am familiar with and connected to the phrase, “The personal is political.” However, as a graduate student who is still developing a pedagogical style, I wonder if the personal should also be pedagogical? Various sociologists that I have met over my graduate career have advised me that emotions and personal experiences do not belong in the classroom. “Just stick to the sociology” is the advice I receive from people who have much more experience teaching than I do. They tell me that I am supposed to be teaching students to be objective, to be neutral, and to rely on data to tell the story of inequality. What happens, however, when pedagogy is painful—when it literally hurts to teach and be taught? How should a professor balance between the emotional experiences generated from the real-world events outside of the classroom with the learning objectives of the classroom?

I wrestled with this question particularly after teaching the course Race and Ethnicity (R&E) at Emory University, a historically white institution (HWI) situated in the South during the fall of 2015. What is the responsibility of the scholar who advances social justice and teaches with the goal of inspiring growth and change? According to Sweet (1998), this kind of pedagogy is one that invites student concerns and demands a fresh approach each time the class is taught. While there are a number of scholarly works that provide tremendous guidance, much of the research comes from the perspective of the faculty and graduate students who teach these courses. What is lacking, however, is the personal perspective of the students taking these courses and the instructor who teaches the material. This paper combines the experiences of student and teacher to describe the personal experiences from both sides of the podium. In what ways can, and should, professors use their own emotions and consider the emotions of the students themselves when designing, organizing, and teaching controversial courses?

To answer this question, I invited a diverse group of former students to provide perspectives, and ultimately, lessons, generated from their individual experiences in my R&E course. There were 38 students in the course that semester. When considering potential coauthors, I looked for social characteristics that reflected the diversity in the classroom and the university. I also made sure to ask students that I would not teach again or who had graduated to reduce the imbalance of power between teacher and student. In the end, I invited seven students to contribute to the article and five responded by submitting their portion. They described themselves in the following manner:

Christina, a black woman

Jennifer, a first-generation Korean American woman

Jonathan, a queer, Latinx man

Jamesetta, a Liberian American woman and child of God

Rocco, a gay, white man

I asked them to introduce themselves, explain why they took the course, discuss their experience in the course, discuss what they wish had been included in the course, and explain what they need from professors when the subject matter is challenging/painful.

This conversation is framed in four parts. First, I summarize the literature related to pedagogical practices deemed radical or rooted in social justice particularly around issues of race. I was searching for, but did not find, articles that would discuss how to address the personal and painful experiences of both student and teacher. Second, I set the historical and social context of the “tumultuous times” that occurred on campus and across the United States. In doing so, I use the students’ own words to describe their personal experiences as well as the emotional pain they felt on campus and in class during a particularly stressful time period. In addition to directly quoting the students, I use their words to describe the campus, classroom, and their experiences. Third, I describe my own personal and emotional experience of the course as a Chicana graduate student. Fourth, I synthesize the student’s suggestions in an effort to contribute to an ongoing conversation about how to reduce the effects of painful pedagogy. Finally, in sharing my own lessons, I discuss strategies for how instructors can connect the emotional, pedagogical, and political experiences to the scholarly research. This article is a collaboration between student and teacher written with the goal of sharing experiences that prompt discussion on the role of emotions and personal experiences in a the classroom of professors committed to social justice.

## Seeking Guidance about Painful Pedagogy

My questions regarding painful pedagogy emerged when I tried to find guidance about dealing with emotional experiences in the classroom. What I discovered was a mixture of various scholarly discussions related to teaching challenging and/or controversial courses that can create conflict, particularly as it relates to race, but no articles that discussed fear, rage, sadness, heartbreak, and other potent emotions. There are also plenty of articles about how faculty and student experience race in the classrooms separately but a dearth of articles that discuss both experiences simultaneously. This conversation, we hope, prompts a deeper exploration of whether or how to manage, discuss, and perhaps even incorporate emotions of both professor and student in the classroom.

Much of the literature that considers personal experiences is deeply rooted in principles of social justice. Teaching styles rooted in social justice had several descriptors, including but not limited to “[making] the personal political,” “scholar activism,” “radical pedagogy,” “critical pedagogy,” and “education by the current event” (Bell and Edmonds 1993; hooks 1994; Sweet 1998; Freire 2000; Wahl et al. 2000). When Sweet (1998) identified forms of radical pedagogy, he isolated a very important value that even his critics shared. Many teachers of sociology, who are committed to some form of social justice, teach purposefully in order to help students understand oppression and to nurture their desire to combat it. I found, however, that many of my students of color were already deeply inspired to identify and confront oppression. As people of color (POCs) and members of historically marginalized groups, they were already painfully aware of oppression and worked every day to combat it. What I could offer, besides validation, was to teach how they could interpret their experiences through sociological theory and empirically rooted analysis. Therefore, while these styles of pedagogy challenge traditional notions of distant, objective, and neutral pedagogy, I was still left wondering what to do with the *emotions* required to deploy those forms of pedagogy. The literature, I found, is largely framed around the ideas that such courses present challenges, controversy, and/or conflicts.

The literature seems to talk around emotions or describes emotional experiences, but very few articles treat it as a central theme or question. For example, Wahl et al. (2000:316) organized a group of racially and ethnically diverse faculty members who engaged in a yearlong workshop to discuss the challenges inherent in teaching a course on race relations. “In these classes,” they write, “we face a conflictual [*sic*] atmosphere that reflects not only our [the United States’] contentious history on matters of race but also several cross-cutting currents that characterize this particular historical moment.” Experiences of oppression, racism, and discrimination are not simply relics of the past but very much the lived experience of the students. These current events often elicit strong emotional responses. While they offer valuable insight in how institutions can manage and support controversial topics, their workshop involved teachers but no students. By no means am I suggesting that the absence of student input makes their suggestions invalid. I am, however, identifying a gap in the literature. Professors and departments are accustomed to reaching “up and across” to fellow colleagues but not necessarily “down and back” to students who are often the recipients of course materials and participants in discussion that are inherently controversial.

Throughout the years, teachers and professors have offered several suggestions for how to overcome a variety of challenges presented in a controversial classroom (Linley 2014; Thorington 2014). Chaisson’s (2004) recounting of her experience teaching critical race theory to white students in an effort to help them understand their own position in the racial hierarchy, individually and collectively, is particularly useful. She describes that her students displayed emotional responses of anger, cognitive distancing, fear of judgment, and even relief and disgust when confronted with white privilege. Her recounting of the students in her course reveals a genuine pain at being taught the reality of race. It seems that learning about white privilege was like exercising a muscle they did not know they had. It hurts at first, but the more it is exercised, the stronger it gets but not without some initial pain. While she masterfully captures the experiences of her students through their writing, she writes very little about her own experiences in teaching, particularly as a woman of color. She also does not write about the students of color in her course for which critical race theory may be an intellectual exercise as well a lived experience.

Other scholars have focused on the experiences of students of color in predominantly white classrooms (Zanolini Morrison 2010; Brunsma, Embrick, and Shin 2016) and white students in racially diverse classrooms (Collett, Kelly, and Sobolewski 2010; Packard 2012). The focus, however, is on the student and not the faculty. For students of color, their experiences range from isolation to validation. They are also more likely to connect the sociological material to their lived experience than their white counterparts (Packard 2012). When describing the experiences of black students at white colleges, Feagin, Vera, and Imani (1996) use the word *agony* as their descriptor. They do not, however, provide guidance on how professors should manage or recognize that agony. They also do not discuss what to do when the professor feels that agony as well. As for white students, much of the literature reveals that the benefits of diversity only comes if and when white students actively engage with students from diverse backgrounds and participate in dialogues with diverse student populations (M. Chang 1999; Gurin et al. 2002).

When it comes to incorporating the personal experiences of students, some scholars challenge teachers to more actively incorporate students’ experiences into the process of learning (Kubal et. al. 2003; Fobes and Kaufman 2008). Students can and should connect their point of view into the curriculum. This requires professors to “decenter” their authority and position in the classroom. Bickel (2006) suggests a co-ownership of the classroom where student and teacher determine the subjects, readings, due dates, page lengths, grading rubrics, and the like. However, surrendering authority when it comes to being a new faculty of color comes with two interesting quandaries for me. First, how do I achieve this balance as a woman of color when my authority and competence may already be under question in the classroom (Hendrix 1998; Balderrama, Teixera, and Valdez 2004; Pittman 2010; Howard-Baptiste 2014)? Second, how do I ask students to design a curriculum when they signed up for my class, presumably, to learn more about this particular issue? For example, students may not know or even request materials about intersectionality, critical race theory, or black feminist thought.

While I am not quite prepared to surrender control as Fobes and Kaufman (2008) suggest, I do agree with their assertion that instructors should require of themselves that which they require of their students. They contend,

If we hope that students are engaged with the course material and with the outside world, then we need to demonstrate what such engagement looks like. We cannot rest on our laurels and rely on what worked well in the past. We need to constantly create and recreate the course based on the students in the classroom, the current state of affairs, and our own development as human beings. (Fobes and Kaufman 2008:29)

While their paper affirms this particular point, what it is still missing is an honest assessment of the emotional toll a course can take on the professor. A course requires more than keeping up with the latest literature and controversies. It also requires acknowledging one’s own position, placement, and experience during tumultuous times. It no doubt requires a delicate balance to be reflexive without making the personal problematic when using experiential methods in the classroom (Grauerholz and Copenhaver 1994).

In order to create a classroom where challenging and emotional topics can be discussed, some professors attempt to create a “safe space” for students to share ideas, thoughts, and feelings. I personally use a modified version of Lynne Weber Cannon’s (1990) “Guidelines for Discussion” for all my courses. I have since discovered, however, that the issue of safe spaces comes with its own set of controversies. On one hand, popular media reports that safe spaces are criticized as too politically correct or as glorified coddling mechanisms for tenderhearted students (Shulevitz 2015; Hartocollis 2016; Furendi 2017). On the other hand, attempts to establish safe spaces in which racial dialogues can be conducted have also been criticized. Antiracist activist Tim Wise (2004:1) has described this practice as “watered down ‘diversity-fests,’ where participants are encouraged to . . . hold hands, sing kumbaya, and feel each other’s existential pain.” Furthermore, the call for safe spaces, he asserts, are more for the benefit of white participants than for POCs. As I think back through my courses, I can understand his point and wonder if that is the kind of environment I am creating.

In response, some scholars encourage the practice of creating “brave spaces.” Brave spaces, as described by Arao and Clemens (2013:141–49) are jointly defined guidelines to engage in difficult conversations with the understanding that it requires disagreement, “strong emotion and rigorous challenge,” a sharing of the “emotional load,” pushing “the boundaries” of comfort zones, and challenging participants in a respectful manner. Brave spaces, as described, are laden with much more emotional language that, according to traditional pedagogy, may not belong in a classroom. Brave spaces also seem to be spaces constructed by and for the benefit of students but not necessarily the professor. Books that encourage brave spaces where “courageous conversations” about race can take place are more instructional for faculty than it is inclusive of their experience (Singleton 2015).

Finally, the emotional experiences and emotional labor carried by professors generally and professors of color specifically helped me to identify my emotions but missed an opportunity to provide strategies to manage them. In studying the experiences of graduate instructors who teach for the first time, Meanwell and Kleiner (2014) found that the graduate students they interviewed described their emotional experience of teaching as both positive and negative. Their subjects used the words *exhausting*, “*a dark and ugly funk*,” *stressful*, and *draining* to describe the emotions they felt as a result of teaching (Meanwell and Kleiner 2014:20).

Through 17 in-depth interviews with women faculty of color teaching and researching at HWIs, Pittman (2010) powerfully captures the emotional experiences that come from gendered racism. Her informants reported having to “be very careful,” explaining, “I can be sensitive as a human being, but I can’t open up totally” (Pittman 2010:188). Another professor described feeling “fearful and angry” when confronted by a white student who accused her of failing to provide a safe space for the white students in her course (Pittman 2010:189). Her study describes feelings of isolation, frustration, and an invisibility that feels sadly familiar.

The emotions of managing white students are eclipsed, however, with the emotional labor required to mentor historically underrepresented students. Julie Shayne (2017) describes emotional labor as “supporting [structurally and institutionally marginalized] students as they experience alienation, marginalization, and trauma, which prevent them from working to their full potential.” This type of emotional labor, studies show, is often uncompensated, unrecognized, and undervalued (Bellas 1999; Wong 2007; Green 2015; Levin et al. 2015; Matthew 2016; Pabon 2016). It is interesting, for example, that during training sessions and faculty meetings I have heard the phrase “I’m not a therapist” in response to working with students experiencing personal challenges. But when it comes to the personal challenges of students of color, the phrase seems to change from therapist to mentor. This discussion of emotional labor is where I found my experience captured outside the classroom. However, it still failed to provide guidance for what to do inside the classroom.

## Tumultuous Times: The Students’ Experience

Throughout history, there have been moments marked by turmoil that signal a significant societal shift. From the murder of Emmett Till to the Los Angeles Revolution, each generation has its moment that inspired social action that pushes back against unjust policies and challenges social, legal, and political inequalities. For my students, it was the fall 2015 events of the University of Missouri that prompted a palpable shift in the energy in my R&E classroom. There, on a national stage, the cries of frustrated, ignored, and marginalized students were projected to the entire nation (if not the world). It was not just the University of Missouri. It was Yale, Duke, Columbia; those cries also poured out of the campus of Emory University. The Black Students of Emory (BSE) movement took root, and a number of students from the course were involved in its organizing. While I was lecturing about historical and contemporary racial injustice, students were confronting it on campus and generating a list of demands (BSE 2015). The political became tremendously personal that particular semester.

The students, without prompting, described how they felt around campus that semester. From depression and hopelessness to anger and frustration, my coauthors described numerous negative and even violent emotions. Jonathan explained that his “brown, queer body” was a “site of colonization . . . a site of trauma, a site of daily violence.” Referring to issues of police brutality, segregation, and deportation, as well as antiblack and anti-immigrant rhetoric, he wrote, “The migraines became more consistent. The depression came in longer episodes.” Other students reported mental and emotional assaults in the form of a social media application (app) called Yik Yak, where students could post comments anonymously. The comments posted to this app were littered with verbally abusive and racist remarks. One student shared with me that as he walked around campus he looked at white students and wondered, “Did you write that? Do you feel that way?” This, if anything, was a clear example of what W. E. B. Du Bois identified as double consciousness: experiencing the world through two sets of eyes simultaneously.

Jamesetta used the phrase “extremely overwhelming” to describe her feelings on campus that semester, saying that it felt as if “a huge *racism outbreak*” emerged that she could not escape. She described walking around campus experiencing microaggressions, prejudice, and racism from students and teachers only to return to her home and witness people dying and suffering all around the world, in schools, and in the streets.

I asked the students to write about why they took the course and to describe their experience of it. Their responses also contained a range of emotions, but most describe immense pain and overwhelming sadness. Jamesetta explains that taking this course during such a trying time for the black community was “devastating.” She recounted that going to class felt like reliving her own ancestral struggle as well as the struggles of her “Latino American, Native American, Asian American brother and sisters.” She described the classroom as “therapeutic but also mentally and emotionally exhausting.”

Christina shared a similar experience. She wrote about the seemingly unending process of having “the race talk” in class, on social media, and in her home. When watching the news, reading books, engaging in academic discourse, and performing her daily activities, she was reminded that “the odds are stacked against black women.” She was reminded that the “American Dream” might never be her reality. She described this process as painful, stressful, and discouraging. She, along with her friends, would wonder if their $200,000 education was worth making only 68 cents to the dollar of a white man. She confessed that she wondered, was it worth it for students of color to try and better themselves if they are only “going to be looked at as inferior on the grand scheme of things?”

Jennifer explained that while she was eager to “learn the language of race scholars,” learning about the history of contemporary racism experienced by Asian American Pacific Islanders (AAPIs) did not make her “feel any better.” She found that despite being labeled a “model minority,” she and members of her community still experience “hellish ridicule and belittlement” but have learned to “take pain and mask it with self-satisfaction.”

Jamesetta further described the pain of learning about racism at an intellectual level while also experiencing personal realizations. “It hurt to realize the prevalence and deep-rooted anti-blackness in modern culture,” she wrote. “It hurt to see the evidence of anti-blackness in everyday mundane things.” While Jamesetta witnessed anti-blackness, Jennifer and Rocco were confronting it within their own communities.

Jennifer explained that it was “absolutely debilitating” to realize how her experiences fit into the race narrative and racial hierarchy. She vividly described how her mother would put sunscreen on her face to avoid being “black as chocolate” while she looked in the mirror longing to be whiter skinned. Describing her AAPI community as both victims and perpetrators, she longed for reconciliation to relieve “the guilt that weighs down hard” for her own complicity in believing, at one time, that black and Latinx students were illegitimate and less skilled. However, she also recognized that the oppression her community experiences, while different, is no less important to consider. She shared, “Police don’t take [Asians] seriously because they don’t understand our accents and politicians consider [Asians] passive constituents whose loyalty belongs to the ‘motherland’ despite being full American citizens.”

Rocco acknowledged that he lived much of his life sheltered in a very wealthy and white part of Orlando, Florida. Even though he joined the course to understand the experiences of others, he struggled to understand his role and responsibility as white, gay male with privilege. While he noticed racism in the gay community, he was also wary of the homophobia within certain racial communities. As he began noticing racism in every aspect of his life, he wrote, “It angered me and inspired me to explore questions regarding the root of the problem.”

Ultimately, it was Jonathan’s description that was the most heartbreaking to read. “Without a doubt,” he wrote, “the course was, at times, a dagger that kept stabbing an open wound.” I knew my students were hurting, but I did not recognize that my course was a source of their pain. In the next section, I describe my own parallel experience teaching the course.

## Tumultuous Times: The Teacher’s Experience

Family, friends, and colleagues tell me that I wear my heart on my sleeve. I love teaching and have been told that, too, is obvious. In my student evaluations, the word that comes up most often is *passionate*. Wearing my heart on my sleeve, however, also means that anyone can tell when I am angry, frustrated, or sad. I try, but fail miserably, to “fix my face.” I am, in a word, emotional. I am also very personal. I share stories of family, partner, and child so regularly that my students inevitably ask to meet them. While I struggle with whether or not these characteristics are stereotypically associated with women and Latina professors, I am beginning to embrace these qualities as strengths rather than liabilities.

The fall of 2015 marked the first time my class would be opened to 40 students. It was the second time I taught the course by myself. When I teach my R&E course, I structure it in the following manner: First, I establish the theoretical foundations on which the course is built discussing critical race theory, racialization, racial formation, the myth of color blindness, intersectionality, and feminist theory. Next, I focus on the sociological, legal, and historical construction of race across each of the different racial groups as well as whites and multiracial Americans. Afterward, I introduce contemporary racial inequalities as they are related to education; crime; lesbian, gay, bisexual, transgender, and queer/questioning plus (LGBTQ+) communities; racial identity politics; and mental health. Each week, I worked diligently to be as inclusive as possible by providing literature on all the different racial and ethnic groups written by a diverse pool of authors. I love teaching this course, and this semester was no different.

However, several events occurred over the summer and into the fall of 2015 that made its way into the classroom. First, there was the brutal death of Freddie Gray in the back of a police truck as well as the suspicious death of Sandra Bland by asphyxiation. Over the summer, the Black Lives Matter movement was growing in magnitude, becoming more organized, and inspiring students to activism. Also in November, a Cook County judge ruled that the dash cam video of 17-year-old Laquan McDonald being shot 16 times by a Chicago police officer should be released to the public. The video was gruesome, heartbreaking, and maddening. Almost simultaneously, students at the University of Missouri, in response to racial incidents on campus, began demanding that the administration confront the racism on campus, provide reparations in the form of free tuition, and divest from for-profit prison companies.

It was during this time that I felt a palpable shift in the energy of the classroom. Some of my students who were very engaged in the course stopped attending class. Other students would visit my office and report feeling stressed, frustrated, and lonely. Others would say things like, “Every time I leave this class, I leave more hopeless than when I came in.” I grew concerned that the course materials were contributing to their sense of hopelessness. During that particular time, they read articles about mass incarceration, lynching in the West, racial profiling, the deaths of Native Americans at the hands of police, and studies debunking the connections between immigration and crime that Trump attempted to make with his “criminals and rapists” comment over the summer.

At one point, I shared with my partner that I lost my students and had no idea how to get them back. All I knew to do was to stay the course and continue teaching the syllabus I had organized. I felt like the course materials wounded my students, especially the ones who were at one time excited about the course. I tried contacting them with no success and hated knowing that they would likely earn a D in my course.

That particular semester, I remember feeling isolated because the members of the beloved community that I had developed in graduate school were in the last year of their program writing their dissertations or had moved away to another city. When I shared my concerns, a faculty member simply told me that I should not allow the events of the semester to change my standards. The students who do not do the work should not be given special consideration especially when there are students who are diligently completing the assignments. I was not their therapist or friend. I was their professor.

I did not know how to explain that I, too, was hurting because they were hurting. I felt as if I could not express that I, too, felt anger, sadness, and hopelessness at the tragic, needless, and unpunished deaths of black men, women, and children at the hands of the police. I could not say that as Trump’s anti-immigrant rhetoric increased, I experienced moments where people would initiate intense conversations with me about why “my people” could not follow the law and would openly tell me that the government needs to send “you people” back to Mexico. I was also given, on numerous occasions, the backhanded compliment and assurance that Trump was not talking about me because I was not like “those people.” It felt as if they had always just stopped short of calling me a “good Mexican.”

As students at the University of Missouri voiced their concerns of racism on campus, my students began to speak openly about the additional pressures, which is supported by the literature, that they felt on campus and in the classroom as first-generation students and students of color (Pascarella et al. 2004; Lundberg et al. 2007; Saenz 2007). In addition to simply being students, they were organizing rallies, attending board-of-trustees meetings, and participating in campus discussions. Rather than punish their activism, I encouraged them to write about it. I explained that they were living the sociology within the readings on racial formation, color blindness, structural inequality, racial identity, and class and wealth inequality. I included questions on the midterm and final that related to contemporary events. I tried to make the classroom a place that reflected the real world. I canceled class in support of a student march and met with students often in and outside of office hours to provide them a place to vent, cry, and feel validated.

This seemed to help the students. Jamesetta shared that as an advocate for social justice, she decided to participate in a locally organized march that ended in the middle of the intersection of a major roadway in front of the university and blocked traffic. “It was risky,” she explains, “but it was necessary to show the world that enough was enough and that the black students at Emory would no longer be silent.” Rocco shared how recognizing institutional racism encouraged him to become involved in social movements and teach-ins on campus associated with Black Lives Matter and the Dakota Access Pipeline. He confessed that he used the course materials to “fact-check” family members and friends who made racist or insensitive remarks.

From participating in a march to correcting a family member, many students do not recognize their actions as an example of the sociological imagination we try so hard to get them to understand. Asking students to connect what happens outside the classroom to what they are learning inside the classroom is exactly the kind of critical thinking and analysis we push them to develop.

Still, I could not shake the feeling that I was missing the mark. I was not giving the students what they needed. It was this feeling that prompted me to include the questions of what they wish I had done differently and what they need from professors who teach challenging courses. Their requests and suggestions are listed in the next section, and my response is in the succeeding section.

## The Students’ Requests and Suggestions

### Recognize the Diversity within Black Communities

Jamesetta shared that I did not include enough materials on the diversity within African American communities in the United States. As a child of two middle-class, immigrant parents from Liberia, she represented the “diasporization” of African immigrants who came to the United States as students and/or resourced individuals (Ette 2011; Arthur, Takougang, and Owusu 2012). Her multiple, intersecting experiences were not reflected in my syllabus despite being represented in the literature on race and immigration (Cross 1991; Waters 1999; Wharton 2008).

Black communities, she explained, reflect as much diversity as Latinx and Asian American communities. They, too, represent different countries, languages, customs, traditions, histories, and experiences. They, too, are wealthy, middle class, successful, and accomplished, and complicate the U.S. racial narrative. These stories, she explained, are not recognized in society and were not reflected in my syllabus. Instead, she learned about the historical roots of racial inequality and their contemporary effects for those individuals whose entire experience is based in the United States. People experience their blackness in similar *and* disparate ways.

### Recognize the Potential Impact of Who Is Teaching the Course

When a POC occupies the role of professor, the responsibilities and implications are clear. This professor, for example, may be the only POC students have encountered in their entire academic career. Christina and Jennifer both shared that, prior to this course, they had never in their entire educational experience had a teacher or professor of color or been taught by fellow member of the AAPI community, respectively. Christina shared that when she was a young girl, she compartmentalized careers by race. She always assumed her education would come from someone who did not look like her. Jonathan explained that taking the course was a “survival tactic” for him to manage life at an HWI and that having a POC teaching the course was empowering and helped him to have a vision of becoming an educator as well. “Students of color,” he contends, “need an ally to let them know that they are not alone and to help [students of color] stay grounded.”

Just as there are unique, unwritten responsibilities for professors of color who teach an R&E course, there are also, according to my coauthors, some very important things a white professor needs to consider. Christiana A., for example, provides an important example of the dangers of not recognizing and/or acknowledging one’s own privilege. She describes an instance when a white professor told a Nigerian student that her personal experience did not make sense because of what he had studied about Yoruba culture. She wrote, “This was frustrating and annoying, and an ineffective way of creating dialogue. Instead, the professor could have affirmed the student’s experience and presented what he knew about Yoruba culture to open a dialogue about a potential explanation for the discrepancy.” When professors acknowledge or are aware of their own privilege, the students explained, it demonstrates humility and promotes healthy and helpful conversation.

### Recognize and Reframe Deficit-model Thinking

Jamesetta affirmed my concern that the course materials were deeply embedded in a deficit model, where POCs embody all the deficits while whites set the standards. She wrote, “After discussing the course with fellow students, we realized that there is a need for courses that teach about the painful past but also celebrates the contributions and successes” of POCs. Professors who teach race, she contends, need to “brighten up” the material with some triumph and success, thereby making the subject matter easier to swallow. She longed for materials that would highlight the resilience of marginalized communities so that being a POC “does not feel like a tragedy when studying history.”

The “white-is-right” narrative dominates our discussions regarding inequality. Whites make more money, own bigger houses, have access to better education, and benefit in ways we are just beginning to measure. Whiteness, however, is not always the ideal. Whiteness is not always the measuring stick that POCs use to measure success. Rocco shared that he and other white students in the course were surprised to hear that, among communities of color, whites were sometimes considered dirty, promiscuous, lazy, academically mediocre, too focused on sports, and classless (Bettie 2000; Espiritu 2001; Jiménez and Horowitz 2013). These readings could enhance the discussion on stereotypes and why these stereotypes do not “stick” on white people in the same way stereotypes seem permanently attached to POCs.

### Practice Rigorous Inclusivity

All of the students reported that moving racial discourse beyond the black/white binary was appreciated. Jamesetta wrote,

[In class] we discussed how other minorities have had similar battles in the world. Instead of just focusing on the Black community, we need to recognize the brothers and sisters that we need to fight alongside. Their stories are not really heard.

Christina echoed the sentiment, explaining that she “wanted to learn about how all cultures work together . . . not just black and white.” Jonathan shared that taking the time and care into making sure the content of the course is inclusive and relevant “makes a difference.”

Rocco explained that by watching documentaries that discussed the portrayal of minorities, he learned how deeply it could impact how people view those groups but also how they see themselves. Recently, after viewing the Disney remake of *Beauty and the Beast*, which featured a gay character with a very small role, he experienced a strong emotional response. “After leaving the theater,” he wrote, “I cried in my car imagining how impactful it would have been to see a gay character when I was a child. I was overcome with emotion thinking about how all the confused little boys and girls would be comforted by this representation.” He shared that positive portrayals of characters from diverse populations can be an enormous tool in educating adults and children from all walks of life.

## Pedagogical and Emotional Pathways for Professors

All of the students’ requests and suggestions challenged me to sit back and determine how I could incorporate their requests in the next iteration of my syllabus. Their suggestions left me both inspired and affirmed. I realized that some of my students felt left out of my syllabus, comforted by and concerned about me as a teacher, frustrated yet inspired to act, and eager to learn more about others. For many of my colleagues who teach R&E courses, much of this may seem obvious. However, as a graduate student who was developing her own expertise and learning scholarly norms, it was not always obvious to me. After much reflection, the following are some of the pedagogical changes I will make and also some strategies for managing my own emotions.

### Incorporate and/or Provide More Literature on African/Caribbean Immigrants, Afro-Latinx Experiences, and the Black Middle Class

While I realize I cannot capture *every* experience, I can, at the very least, include more articles on the experiences of the children of African and Caribbean immigrants. The narrative and social history of African Americans in the United States is not their story. Like Afro-Latinx individuals, they occupy a position where race and ethnicity collide. They are raced as black in America, but everything about their homes, their language, their food, and their life is Nigerian, Ghanaian, Haitian, or Guyanese, to name a few. Those experiences cannot and should not be erased in the racialization process.

In the beginning of the semester, I ask students to write a paper in response to the following question: What race are you? How do you know?^2^ I have read numerous essays that contain the sentence, “I didn’t know I was black until I came to the states.” In addition to being supported by the literature, this disconnect has been the subject of intense debate within black communities. Henry Louis Gates and Lani Guinier, for example, debated how the admissions of African and Caribbean students have become a substitute for accepting African American students (Rimer and Arenson 2004). I should discuss this issue and incorporate those identities as well.

I also need to include the experiences of middle- and upper-class POCs. There is a wealth of literature on the black middle and upper classes (Graham 1999; Lareau 2003; Lacy 2007; Harris 2013; Lacy 2015), the Latinx middle class (Vallejo 2015), and the Asian American middle class (Ngai 2010; Lee and Zhou 2014). From the Talented Tenth to strategic assimilation, the literature beautifully captures the experiences those who have “made it” economically but have yet to escape racial and/or ethnic discrimination and prejudice. There are classic texts from which I can have them read an excerpt, including *Black Bourgeoisie* (Frazier 1957), *Black Wealth/White Wealth* (Oliver and Shapiro 1995), *The Hidden Cost of Being African American* (Shapiro 2004), or *Whistling Vivaldi* (Steele 2010). The challenge, of course, is that there are so many readings but so little time. Still, I can and will create a list of readings for students to consider after the course. This list, at the very least, will signal to students my understanding of the complexity and ever-expanding scholarship on topics related to race and ethnicity. It can also encourage them to find pathways for their own sociological journey.

### Pedagogy Requires Reflexivity

Jonathan correctly identifies, and the literature supports, that the task of mentoring students of color will mostly fall on the shoulders of professors of color. Numerous scholars identify the challenges of uncompensated personal work that comes with being a scholar of color to whom historically marginalized students are drawn. I regularly find myself mentoring more undergraduate students of color than my professors mentor graduate students. It is both a privilege and a challenge. On one hand, I remember what it is like to be mentored by faculty of color. I appreciate the time and guidance they gave to me and consider it an opportunity to “pay it forward” today. On the other hand, I, myself, am a student who wants and needs to finish my own dissertation. There are times when I need to be selfish and pursue my own projects.

Furthermore, if I am choosing to wear my heart on my sleeve, I need to think critically about the how, when, and why I do it. Looking back, there were times when, in addition to the assigned articles, I knew that a personal story about intersectionality would help them understand the concept and think critically about how to incorporate it into their research. Sometimes it helped with starting difficult conversations, such as the silence within communities of color regarding LGBTQ+ identities. The class discussion seemed to open up after I shared about my how my extended family never acknowledged that a much-loved uncle was gay. When I tell the students that my family just never talked about it, it provides a space for us to discuss research from *Invisible Families* (Moore 2011) or research on the acceptance of Two-Spiritness within Native Americans (Adams and Phillips 2009). I did not always share personal stories. There were also plenty of times when I held back to allow for the pregnant silence that often accompanies critical thinking. Reflecting on this course helped me to understand that there is a balance to be struck and a strategy I must develop.

Finally, being a woman of color does not exempt me from recognizing my own privilege and practicing humility. As a heterosexual, able-bodied, upper-middle-class Chicana whose racial identity can bounce between the black/white binary of race depending on the weather and circumstance, I need to be keenly aware of the privileges I possess. A student’s experience can inform, not be excluded from, my expertise. I do not want to miss out on meaningful dialogue because a student’s story is not “captured in the data.” As an academic and intellectual developing an expertise on race and ethnicity, I can and should remember that I, too, am always learning and possess several blind spots. This requires a great deal of personal reflection about and painful realizations of my own conscious and unconscious biases.

### Reduce and Confront Deficit Thinking

I share with my students that sociologists tend to study the catastrophic. As a result, I need to be mindful on how the assigned readings, documentaries, and class discussions could add to their despair. For example, I screen four documentaries about race and Hollywood to explore the roots of stereotypes and controlling images (Collins 2009; Golash-Boza 2015). Those documentaries are *Ethnic Notions* (Riggs [1986] 2004), *The Bronze Screen* (De Los Santos, Dominguez, and De Jesus 2002), *Reel Injun* (Diamond 2010), and *The Slanted Screen* (Adachi [2006] 2010) or *Slaying the Dragon Reloaded* (Gee 2011). They are amazing documentaries that I have used on numerous occasions to demonstrate the role of media in constructing controlling images of black, Latinx, Native American, and Asian American communities. However, I realized that I never warned the students about some of the images, dialogue, and commentary in the documentaries. For example, I have seen *Ethnic Notions* at least 25 times at various points in my professional and academic career. Every time I watch it, there is a terribly racist song that, unfortunately, gets stuck in my head. It always takes me a few days to get it out, and I generally need a day to decompress. If, even after watching it numerous times, I need a day, then students may need it as well. In the future, I will warn students and leave more time for discussion.

Second, I need to reconsider and reexamine the assigned readings to determine if they add theoretical and empirical value and advance the discussion or if I am simply relentlessly repeating the same point. For example, I assigned Ronald Takaki’s (2008) *A Different Mirror* to examine the social histories of each racial group. Specifically, I assign three chapters for each group. Having reread the material, however, I realize that one chapter is truly enough. Each chapter, while rich in detail and imagery, is an assault on the imagination. From Andrew Jackson’s slaughter of Native Americans to the frighteningly detailed descriptions of lynching, the story of race in the United States is vicious and brutal. But do students really need one chapter after another of this brutality? Instead of making the extra chapters required reading, I will suggest it as supplementary materials.

Finally, offering material that is “happy and light” is a bit more difficult when teaching about the historical, structural, and legal realities of racism, prejudice, and discrimination. There is, however, no shortage of materials where the marginalized are also the empowered, the strong, and the victorious. For evidence, I turn to ethnic studies scholars and historians. Laura Pulido’s (2006) book, *Black, Brown, Yellow, and Left*, for example, provides excellent examples of how communities of color supported, encouraged, and inspired one another toward activism. Scott Kurashige’s (2008) book, *The Shifting Grounds of Race*, describes the little-known connections between black and Japanese American communities in Los Angeles during World War II. Mark Brilliant’s (2010) book, *The Color of America Has Changed*, contains excellent chapters that explore interracial coalitions during times of civil rights reform between 1941 and 1978.^3^ Furthermore, there are numerous documentaries from organizations such as Women Make Movies, the Center for Asian American Media, and California Newsreel that showcase these social movements and experiences.

### Continue to Practice Rigorous Inclusivity in Informed Ways

The goals of rigorous inclusivity is to inspire students to see how they can and do contribute to race and ethnic relations and identify opportunities for coalition building across communities of color. At the beginning of the course, I make it clear that I am not putting an equal sign (=) between the experiences of POCs. I am, however, using the mathematical symbol for simile (≈) to demonstrate how oppression works across and through groups in very similar, yet complicated, ways. For example, when I teach about school desegregation efforts, I discuss not only *Brown v. Board of Education* but also cases involving Chinese American plaintiffs (*Tape v. Hurley* and *Gong Lum v. Rice*), Native American plaintiffs (*Piper v. Big Pine* and *McMillan v. School Committee*), and Mexican American plaintiffs (*Mendez v. Westminster* and *Gonzales v. Sheely*). Expanding the discussion to include Asian American, Indigenous, and Latinx experiences not only encourages students to recognize that activism is not just black/white but also allows me to have a more theoretically informed discussion about the U.S. racial hierarchy via Asian critical race theory, tribal critical race theory, and Latinx critical race theory (R. Chang 1993; Haney Lopez 1997; Brayboy 2006; Matsuda 2010; Aparicio 2016).

### Give Oneself Permission and Allow Space to Feel Pain

In his review on the sociology of emotions, Bericat (2016:495) explains that the tasks in studying the sociology of emotions is “studying the social nature of emotions and studying the emotional nature of social reality.” Within that fall R&E classroom, these five students and I witnessed the emotional nature of social reality. In writing this article, we identified the social nature of emotions between student and teacher. As a result, I learned that it might be okay to admit that the day after the 2016 election I, too, cried along with my students, particularly those who were undocumented. I recognize that my tears did not just represent my concern for them. They came from a place that is painfully familiar with the fear of deportation within mixed-status families.

According to the groundbreaking and subsequent work of Arlie R. Hochschild (1979, 1983), I understand that I, too, engage in “emotion work” full of unwritten “feeling rules.” As a result, I also needed to allow space to feel the pain. That space can come in many forms, such as physical activity, meditation, or taking up a personally fulfilling hobby. For me, however, that space became therapy. As a Chicana who grew up being taught to keep everything *en la familia*,^4^ sitting with someone and telling her my deepest, darkest feelings and thoughts felt like a transgression and betrayal at first. Therapy, I had been taught, was for weak, white women. I, on the other hand, had come from a long line of *strong* women who overcame enormous odds and personal tragedies to provide me with the opportunities I now enjoy. In the grand scheme of things, my feelings were nothing compared to the poverty, punishment, and pain my family endured.

Needless to say, it was a big step for me to sift through therapists who found me “fascinating” where I became their personal race-and-culture expert instead of a client. I realized it was okay to ask for their experience with women of color *before* I scheduled an appointment in order to find my own personal brave space.

## Conclusion

This is by no means an exhaustive list of recommendations and is not a substitute for conducting rigorous studies on student learning and/or suggestions for such courses. There is also no one “best” strategy for managing emotions in the classroom. This conversation represents the reflections of students and their professor after a particularly painful semester. I am sure these students completed a course evaluation indicating on a Likert scale whether or not “the instructor explained specific concepts relevant to the course.” However, asking these five students to write about their personal and emotional experience of the course challenged me to parlay their suggestions into the transformative pedagogy that critical consciousness demands. Students need to see their experiences reflected in the syllabus and in the course materials to educate and inspire them and fuel their desire for social change.

With the divisive 2016 presidential election, there does not appear to be an end or reprieve from tumultuous times that sometimes drip and oftentimes flood into our classrooms. This administration provides plenty of material to analyze during a course on race, ethnicity, and immigration. Frankly, it provides plenty of material for any sociology course. Acknowledging the emotional toll such discord may have on self and student is a valuable process in which to engage. Furthermore, engaging former students in a postcourse discussion is another opportunity to identify one’s own blind spots, particularly in an area of expertise. In closing, this collaboration helped me to realize that, while I cannot control painful situations, I can work to transform personal pain into meaningful pedagogy.

## Notes

1. A Muxerista or Mujerista is a Chicana/Latina who is committed to social justice. For a more detailed explanation, see Tijerina Revilla (2004).

2. An assignment borrowed from Dr. Amanda Lewis, professor of African American studies, and University of Illinois–Chicago.

3. While any book in its entirety would be ideal, I would recommend chapters 4 and 6 from Pulido’s (2006) book, chapters 7 and 10 from Kurashige’s (2008) book, and chapters 1 and 6 from Brilliant’s (2010) book.

4. “Within the family.”

## References

[bibr1-0092055X17754120] Adachi Jeff . [2006] 2010. The Slanted Screen: Asian Men in Film and Television. DVD. Hamilton, NJ: Films for Humanities and Sciences.

[bibr2-0092055X17754120] Adams Heather L. Phillips Layli . 2009. “Ethnic Related Variations from the Cass Model of Homosexual Identity Formation: The Experiences of Two-Spirit, Lesbian, and Gay Native Americans.” Journal of Homosexuality 56(7):959–76. 19802764 10.1080/00918360903187895

[bibr3-0092055X17754120] Aparicio Frances R. 2016. “Not Fully Boricuas: Puerto Rican Intralatino/as in Chicago.” Centro Journal 28(2):154–78.

[bibr4-0092055X17754120] Arao Brian Clemens Kristi . 2013. “From Safe Spaces to Brave Spaces: A New Way to Frame Dialogue around Diversity and Social Justice.” Pp. 135–50 in From the Art of Effective Facilitation, edited by Landreman L. M. Sterling, VA: Stylus.

[bibr5-0092055X17754120] Arthur John A. Takougang Joseph Owusu Thomas . 2012. Africans in Global Migration: Searching for Promised Lands. Lanham, MD: Lexington Books.

[bibr6-0092055X17754120] Balderrama Maria Teixera Mary T. Valdez Elsa . 2004. “Una Lucha de Fronteras (A Struggle of Borders): Women of Color in the Academy.” Race, Gender, Class 11(4):135–54.

[bibr7-0092055X17754120] Bell Derrick A. Edmonds Erin . 1993. “Students as Teachers, Teachers as Learners.” Michigan Law Review 91(8):2025–52.

[bibr8-0092055X17754120] Bellas Marcia L. 1999. “Emotional Labor in Academia: The Case of Professor.” Annals of the American Academy of Political and Social Science 561:96–110.

[bibr9-0092055X17754120] Bericat Eduardo . 2016. “The Sociology of Emotions: Four Decades of Progress.” Current Sociology 64(3):491–513.

[bibr10-0092055X17754120] Bettie Julie . 2000. “Women without Class: Chicas, Cholas, Trash, and the Presence/Absence of Class Identity.” Signs 26(1):1–35.

[bibr11-0092055X17754120] Bickel Christopher . 2006. “Cultivating Orchids: Promoting Democracy in the Classroom.” Pp. 93–107 in Critical Pedagogy in the Classroom, edited by Kaufman P. Washington, DC: American Sociological Association.

[bibr12-0092055X17754120] Black Students of Emory. 2015. “Black Students at Emory: List of Demands.” Emory Wheel. Retrieved August 1, 2017 (http://emorywheel.com/black-students-at-emory-list-of-demands/).

[bibr13-0092055X17754120] Brayboy Bryan McKinley Jones . 2006. “Toward a Tribal Critical Race Theory in Education.” Urban Review 37(5):425–46.

[bibr14-0092055X17754120] Brilliant Mark . 2010. The Color of America Has Changed: How Racial Diversity Shaped Civil Rights Reform in California, 1941–1978. New York: Oxford University Press.

[bibr15-0092055X17754120] Brunsma David L. Embrick David G. Shin Jean H. 2016. “Graduate Students of Color: Race, Racism, and Mentoring in the White Waters of Academia.” Sociology of Race and Ethnicity 3(1):1–13.

[bibr16-0092055X17754120] Chaisson Reba L. 2004. “A Crack in the Door: Critical Race Theory in Practice at a Predominantly White Institution.” Teaching Sociology 32(4):345–57.

[bibr17-0092055X17754120] Chang Mitchell . 1999. “Does Racial Diversity Matter? The Educational Impact of a Racially Diverse Undergraduate Population.” Journal of College Student Development 40(4):377–95.

[bibr18-0092055X17754120] Chang Robert S. 1993. “Toward an Asian American Legal Scholarship: Critical Race Theory, Post-structuralism, and Narrative Space.” California Law Review 81(5): 1241–1323.

[bibr19-0092055X17754120] Collett Jessica L. Kelly Sean Sobolewski Curt . 2010. “Using *Remember the Titans* to Teach Theories of Conflict Reduction.” Teaching Sociology 38(3):258–66.

[bibr20-0092055X17754120] Collins Patricia H. 2009. Black Feminist Thought. New York: Routledge Classics.

[bibr21-0092055X17754120] Cross William E. 1991. Shades of Black: Diversity in African-American Identity. Philadelphia: Temple University Press.

[bibr22-0092055X17754120] De Los Santos Susan Racho Dominguez Alberto De Jesus Wanda . 2002. The Bronze Screen: 100 Years of the Latino Image in Hollywood Cinema. DVD. Chicago: Questar.

[bibr23-0092055X17754120] Diamond Neil . 2010. Reel Injun: On the Trail of the Hollywood Indian. DVD. New York: Lorber Films.

[bibr24-0092055X17754120] Espiritu Yen Le . 2001. “We Don’t Sleep Around Like White Girls Do”: Family, Culture, and Gender in Filipina American Lives.” Signs 26(2):415–40.

[bibr25-0092055X17754120] Ette Ezekial Umo . 2011. Nigerian Immigrants in the United States: Race, Identity, and Acculturation. Lanham, MD: Lexington Books.

[bibr26-0092055X17754120] Feagin Joe Vera Hernan Imani Nikitha . 1996. The Agony of Education: Black Students at White Colleges and Universities. New York City: Routledge.

[bibr27-0092055X17754120] Fobes Catherine Kaufman Peter . 2008. “Critical Pedagogy in the Sociology Classroom: Challenges and Concerns.” Teaching Sociology 36(1):26–33.

[bibr28-0092055X17754120] Frazier E. Franklin . 1957. Black Bourgeoisie. New York: Free Press.

[bibr29-0092055X17754120] Freire Pablo . 2000. Pedagogy of the Oppressed. London: Continuum International Group.

[bibr30-0092055X17754120] Furendi Frank . 2017. “Op-ed: Campuses Are Breaking Apart into ‘Safe Spaces.’” Los Angeles Times, January 5. Retrieved July 10, 2017 http://www.latimes.com/opinion/op-ed/la-oe-furedi-safe-space-20170105-story.html

[bibr31-0092055X17754120] Gee Deborah . 2011. Slaying the Dragon Reloaded. DVD. San Francisco: Asian Women United of California.

[bibr32-0092055X17754120] Golash-Boza Tanya Maria . 2015. Race and Racisms: A Critical Approach. New York: Oxford University Press.

[bibr33-0092055X17754120] Graham Lawrence Otis . 1999. Our Kind of People: Inside America’s Black Upper Class. New York: HarperCollins.

[bibr34-0092055X17754120] Grauerholz Elizabeth Copenhaver Stacey . 1994. “When the Personal Becomes Problematic: The Ethics of Using Experiential Teaching Methods.” Teaching Sociology 22(4):319–27.

[bibr35-0092055X17754120] Green Myra . 2015. “Thanks for Listening.” Chronicle of Higher Education, October 19. Retrieved December 26, 2017 http://www.chronicle.com/article/Thanks-for-Listening/233825

[bibr36-0092055X17754120] Gurin Patricia Dey Eric Hurtado Sylvia Gurin Gerald . 2002. “Diversity and Higher Education: Theory and Impact on Educational Outcomes.” Harvard Educational Review 72(3):330–67.

[bibr37-0092055X17754120] Haney López Ian F. 1997. “Race, Ethnicity, Erasure: The Salience of Race to LatCrit Theory.” California Law Review 85(5):1143–1211.

[bibr38-0092055X17754120] Harris Cherise A. 2013. The Cosby Cohort: Blessings and Burdens of Growing Up Black Middle Class. Lanham, MD: Rowman & Littlefield.

[bibr39-0092055X17754120] Hartocollis Anemona . 2016. “On Campus, Trump Fans Say They Need ‘Safe Spaces.’” New York Times, December 8. Retrieved July 10, 2017 (https://www.nytimes.com/2016/12/08/us/politics/political-divide-on-campuses-hardens-after-trumps-victory.html).

[bibr40-0092055X17754120] Hendrix Katherine G. 1998. “Student Perceptions of the Influence of Race on Professor Credibility.” Journal of Black Studies 28(6):738–64.

[bibr41-0092055X17754120] Hochschild Arlie Russell . 1979. “Emotion Work, Feeling Rules, and Social Structure.” American Journal of Sociology 85(3):551–75.

[bibr42-0092055X17754120] Hochschild Arlie Russell . 1983. The Managed Heart: The Commercialization of Human Feeling. Berkeley: University of California Press.

[bibr43-0092055X17754120] hooks bell . 1994. Teaching to Transgress: Education as the Practice of Freedom. New York: Routledge.

[bibr44-0092055X17754120] Howard-Baptiste Shewanee D. 2014. “Artic Space, Lonely Place: ‘Mammy Moments’ in Higher Education.” Urban Review 46(4):764–82.

[bibr45-0092055X17754120] Jiménez Tomás R. Horowitz Adam L. 2013. “When White Is Just Alright: How Immigrants Redefine Achievement and Reconfigure the Ethnoracial Hierarchy.” American Sociological Review 78(5): 849–71.

[bibr46-0092055X17754120] Kubal Timothy Meyler Deanna Stone Rosalie Torres Mauney Teelyn T. 2003. “Teaching Diversity and Learning Outcomes: Bringing Lived Experience into the Classroom.” Teaching Sociology 31(4):441–55.

[bibr47-0092055X17754120] Kurashige Scott . 2008. The Shifting Grounds of Race: Black and Japanese Americans in the Making of Multiethnic Los Angeles. Princeton, NJ: Princeton University Press.

[bibr48-0092055X17754120] Lacy Karyn . 2007. Blue-chip Black: Race, Class, and Status in the New Black Middle Class. Berkeley: University of California Press.

[bibr49-0092055X17754120] Lacy Karyn . 2015. “Race, Privilege and the Growing Class Divide.” Ethnic and Racial Studies 38(8): 1246–49.

[bibr50-0092055X17754120] Lareau Annette . 2003. Unequal Childhoods: Class, Race and Family Life. Berkeley: University of California Press.

[bibr51-0092055X17754120] Lee Jennifer Zhou Min . 2014. “The Success Frame and Achievement Paradox: The Costs and Consequences for Asian Americans.” Race and Social Problems 6(1):38–55.

[bibr52-0092055X17754120] Levin John S. Jackson-Boothby Adam Haberler Zachery Walker Laurencia . 2015. “Dangerous Work: Improving Conditions for Faculty of Color in the Community College.” Community College Journal of Research and Practice 19:1–13.

[bibr53-0092055X17754120] Linley Jodi . 2014. “White Women Getting Real about Race: Their Stories about What They Learned Teaching in Diverse Classrooms.” NASPA Journal about Women in Higher Education 7(2):248–51.

[bibr54-0092055X17754120] Lundberg Carol A. Schreiner Laurie A. Hovaguimian Kristin Miller Sharyn Slavin . 2007. “First-generation Status and Student Race/Ethnicity as Distinct Predictors of Student Involvement and Learning.” NASPA Journal 44(1):57–83.

[bibr55-0092055X17754120] Matsuda Mari . 2010. “We Will Not Be Used: Are Asian Americas the Racial Bourgeoisie?” Pp. 558–64 in Asian American Studies Now, edited by Chen T. Wu J. Y.-W. S. New Brunswick, NJ: Rutgers University Press.

[bibr56-0092055X17754120] Matthew Patricia A. 2016. “What Is Faculty Diversity Worth to a University?” Atlantic, November 23. Retrieved January 27, 2017 (https://www.theatlantic.com/education/archive/2016/11/what-is-faculty-diversity-worth-to-a-university/508334/).

[bibr57-0092055X17754120] Meanwell Emily Kleiner Sibyl . 2014. “The Emotional Experience of First-time Teaching: Reflections from Graduate Instructors, 1997–2006.” Teaching Sociology 42(1):17–27.

[bibr58-0092055X17754120] Moore Mignon . 2011. Invisible Families: Gay Identities, Relationships, and Motherhood among Black Women. Berkeley: University of California Press.

[bibr59-0092055X17754120] Ngai Mae . 2010. The Lucky Ones. New York: Houghton Mifflin Harcourt.

[bibr60-0092055X17754120] Oliver Melvin L. Shapiro Thomas M. 1995. Black Wealth/White Wealth: A New Perspective on Racial Inequality. New York: Routledge.

[bibr61-0092055X17754120] Pabon Amber . 2016. “Waiting for Black Superman.” Urban Education 51(8):915–39.

[bibr62-0092055X17754120] Packard Josh . 2011. “The Impact of Racial Diversity in the Classroom: Activating the Sociological Imagination.” Teaching Sociology 41(2):144–58.

[bibr63-0092055X17754120] Pascarella Earnest T. Pierson Christopher T. Wolniak Gregory C. Terenzini Patrick T. 2004. “First-generation College Students: Additional Evidence on College Experiences and Outcomes.” Journal of Higher Education 75(3):249–84.

[bibr64-0092055X17754120] Pittman Chavella T. 2010. “Race and Gender Oppression in the Classroom: The Experiences of Women Faculty of Color with White Male Students.” Teaching Sociology 38(3):183–96.

[bibr65-0092055X17754120] Pulido Laura . 2006. Black, Brown, Yellow, and Left: Radical Activism in Los Angeles. Berkeley: University of California Press.

[bibr66-0092055X17754120] Riggs Marlon . [1986] 2004. Ethnic Notions. DVD. San Francisco: California Newsreel.

[bibr67-0092055X17754120] Rimer Sara Arenson Karen W. 2004. “Top Colleges Take More Blacks, but Which Ones?” New York Times, June 24. Retrieved January 27, 2017 (http://www.nytimes.com/2004/06/24/us/top-colleges-take-more-blacks-but-which-ones.html?_r=0).

[bibr68-0092055X17754120] Saenz Victor B. 2007. “First in My Family: A Profile of First-generation College Students at Four-year Institutions since 1971.” Los Angeles: Higher Education Research Institute, University of California–Los Angeles.

[bibr69-0092055X17754120] Shapiro Thomas M. 2004. The Hidden Cost of Being African American: How Wealth Perpetuates Inequality. New York: Oxford University Press.

[bibr70-0092055X17754120] Shayne Julie . 2017. “Recognizing Emotional Labor in Academe.” Inside Higher Ed. Retrieved September 15, 2017 (https://www.insidehighered.com/advice/2017/09/15/importance-recognizing-faculty-their-emotional-support-students-essay).

[bibr71-0092055X17754120] Shulevitz Judith . 2015. “In College and Hiding from Scary Ideas” New York Times Sunday Review, March 21. Retrieved July 10, 2017 (https://www.nytimes.com/2015/03/22/opinion/sunday/judith-shulevitz-hiding-from-scary-ideas.html).

[bibr72-0092055X17754120] Singleton Glenn E. 2015. Courageous Conversations about Race: A Field Guide for Achieving Equity in Schools. Thousand Oaks, CA: Corwin.

[bibr73-0092055X17754120] Steele Claude M. 2010. Whistling Vivaldi: How Stereotypes Affect Us and What We Can Do. New York: Norton.

[bibr74-0092055X17754120] Sweet Stephen . 1998. “Practicing Radical Pedagogy: Balancing Ideals with Institutional Constraints.” Teaching Sociology 26(2):100–11.

[bibr75-0092055X17754120] Takaki Ronald . 2008. A Different Mirror: A History of Multicultural America. New York: Back Bay Books/Little, Brown.

[bibr76-0092055X17754120] Thorington Springer Jennifer . 2014. “What’s Post Racial Discourse Got to Do With It? Obama and the Implications for Multiculturalism in College Classrooms.” Journal of Scholarship and Teaching 14(3):1–15.

[bibr77-0092055X17754120] Tijerina Revilla Anita . 2004. “Muxerista Pedagogy: Raza Womyn Teaching Social Justice through Student Activism.” High School Journal 87(4):80–94.

[bibr78-0092055X17754120] Vallejo Jody A. 2015. “Leveling the Playing Field: Patterns of Ethnic Philanthropy among Los Angeles’ Middle- and Upper-class Latino Entrepreneurs.” Ethnic and Racial Studies 38(1):125–40.

[bibr79-0092055X17754120] Wahl Ana-Maria Perez Eduardo T. Jo Deegan Mary Sanchez Thomas W. Applegate Cheryl . 2000. “The Controversial Classroom: Institutional Resources and Pedagogical Strategies for a Race Relations Course.” Teaching Sociology 28(4):316–32.

[bibr80-0092055X17754120] Waters Mary C. 1999. Black Identities: West Indian Immigrant Dreams and American Realities. Cambridge, MA: Harvard University Press.

[bibr81-0092055X17754120] Weber Cannon Lynn . 1990. “Fostering Positive Race, Class, and Gender Dynamics in the Classroom.” Women’s Studies Quarterly 18(1/2):126–34.

[bibr82-0092055X17754120] Wharton Felicia . 2008. “The Identity Transformation Process of the West Indian College Student.” Presented at the Adult Education Research Conference, June 5–7, St. Louis, MO.

[bibr83-0092055X17754120] Wise Tim . 2004. “No Such Place as Safe: The Trouble with White Anti-racism.” Tim Wise Blog. Retrieved September 30, 2017 (http://www.timwise.org/2004/07/no-such-place-as-safe-the-trouble-with-white-anti-racism/).

[bibr84-0092055X17754120] Wong Kathleen . 2007. “Emotional Labor of Diversity Work: Women of Color Faculty in Predominantly White Institutions.” Dissertation. Retrieved from ProQuest Dissertations and Theses Database, 304896286.

[bibr85-0092055X17754120] Zanolini Morrison Gina . 2010. “Students of Color at a Predominantly White University.” Journal of Black Studies 40(5):987–1015.

